# Stress Waves and Characteristics of Zigzag and Armchair Silicene Nanoribbons

**DOI:** 10.3390/nano6070120

**Published:** 2016-06-24

**Authors:** Yu-Cheng Fan, Te-Hua Fang, Tao-Hsing Chen

**Affiliations:** Department of Mechanical Engineering, National Kaohsiung University of Applied Sciences, Kaohsiung 807, Taiwan; poiuytrewq0928@yahoo.com.tw (Y.-C.F.); thchen@kuas.edu.tw (T.-H.C.)

**Keywords:** silicone, fracture, wrinklons, effective modulus

## Abstract

The mechanical properties of silicene nanostructures subject to tensile loading were studied via a molecular dynamics (MD) simulation. The effects of temperature on Young’s modulus and the fracture strain of silicene with armchair and zigzag types were examined. The maximum in-plane stress and the corresponding critical strain of the armchair and the zigzag silicene sheets at 300 K were 8.85 and 10.62, and 0.187 and 0.244 N/m, respectively. The in-plane stresses of the silicene sheet in the armchair direction at the temperatures of 300, 400, 500, and 600 K were 8.85, 8.50, 8.26, and 7.79 N/m, respectively. The in-plane stresses of the silicene sheet in the zigzag direction at the temperatures of 300, 400, 500, and 600 K were 10.62, 9.92, 9.64, and 9.27 N/m, respectively. The improved mechanical properties can be calculated in a silicene sheet yielded in the zigzag direction compared with the tensile loading in the armchair direction. The wrinklons and waves were observed at the shear band across the center zone of the silicene sheet. These results provide useful information about the mechanical and fracture behaviors of silicene for engineering applications.

## 1. Introduction

Since single atomic layer graphene sheets were fabricated in 2004, two-dimensional (2D) materials have received much increasing attention [1]. Recently, single-layer silicon, or silicene, has been successfully prepared and has become a new focus of engineering and scientific research [2,3]. Silicene has attracted great attention because of its excellent physical and electrical properties [4]. However, compared with graphene, boron nitride, monolayer MoS_2_, and other 2D materials, silicene has a low elastic modulus and mechanical strength, which may affect its applications to sensors and devices [5].

Yang et al. [6] found the ideal strengths for uniaxial tension and armchair uniaxial tension based on ab initio calculation. Zhang et al. [7] studied novel finite metal endohedral silicene-like silicon nanotubes with the density functional theory and found that their structural stability increase with an increasing tube length. Li et al. [8] studied the geometrical structures and electronic properties of the armchair- and zigzag-edge silicene nanoribbons that the termination with oxygen, and the hydroxyl-group was investigated using the first-principles method.

In order to understand the mechanics and the interactions of nanomaterials, the simulation method capable of describing the process within the nanomaterials is indeed required. It is worth noting that a molecular dynamics (MD) simulation is capable of accurately describing the mechanical processes of materials in a nanoscale [9,10,11,12]. For instance, Ansari et al. [13] observed that bulk modulus was strongly size-dependent and decreased when increasing the length of silicene nanosheets under uniaxial and biaxial tension using molecular dynamics. Ince and Erkoc [14] found out the effect of increasing the width of a silicene nanoribbon, depending also on the temperature and the presence or absence of boundaries using molecular dynamics simulations. Roman and Cranford [15] found that silicene was relatively weaker than graphene in terms of stiffness, but more rigid when being subject to bending due to its slightly buckled molecular geometry.

In this study, a MD simulation was carried out to study the mechanical properties of the silicene nanostructure at different temperatures subject to tensile loading. Young’s modulus, the fracture strain, and the strain energy of the nanosheets in the armchair and the zigzag directions were explored. We further focused on the mechanical characterization of silicene sheets with crack defects.

## 2. Simulation Method

The Tersoff potential [16] is used for modeling the interaction between silicon atoms. The constant-temperature molecular-dynamics simulations are performed using a velocity scaling thermostat for the temperature control. The silicene model simulation is equilibrated for 20 ps in the canonical ensemble. The simulation time step of 1 fs is employed. Table 1 shows the lattice parameters and the geometric structure of the silicene at equilibrium state. For comparison, Table 1 also includes previous studies of MD and density functional theory (DFT) calculations [17,18,19,20]. In this study, the angle between neighboring bonds was taken as θ_0_ = 117.98° and the bond length *r*_0_ = 2.32 Å. Hence, the lattice constant, *d*_0_, and the buckling height, *D*_0_, could be calculated as 4.00 Å and 0.38 Å, respectively.

The preface armchair and zigzag silicene sheets have the dimensions, in (width) × (length), of 49.9 nm × 56.0 nm and 49.9 nm × 56.1 nm in an approximately squared shape. The armchair and zigzag silicene sheets contain 39,852 and 39,744 atoms. Two layers of silicon atoms on the left and the right, about 3 nm, are fixed in the length direction. The fixed layers are set to move during tensile loading along the y-direction. To study the deformation behavior of the silicene under tensile loading, a positive displacement with a stretch rate of 10 m/s in the lateral directions is applied to the atoms on both the left and the right edges of the silicene sheet.

The linear slope of the in-plane stress $\sigma_{in-plane}$ and the strain ε can be defined as the effective modulus *E* as follows:

(1) $$E=\frac{\partial \sigma_{in-plane}}{\partial \varepsilon }$$

The in-plane stiffness is calculated by using a linear fit of the stress–strain slope with a ranged tensile strain of 0.04–0.08. Silicene can be used for gas absorption or separation. Therefore, the temperature effects on the tensile property of the silicene were examined at different temperatures of 300, 400, 500, and 600 K. Two chiral types of armchair and zigzag on the fracture characteristics of the silicene were also studied.

## 3. Results and Discussion

**Figure 1:**
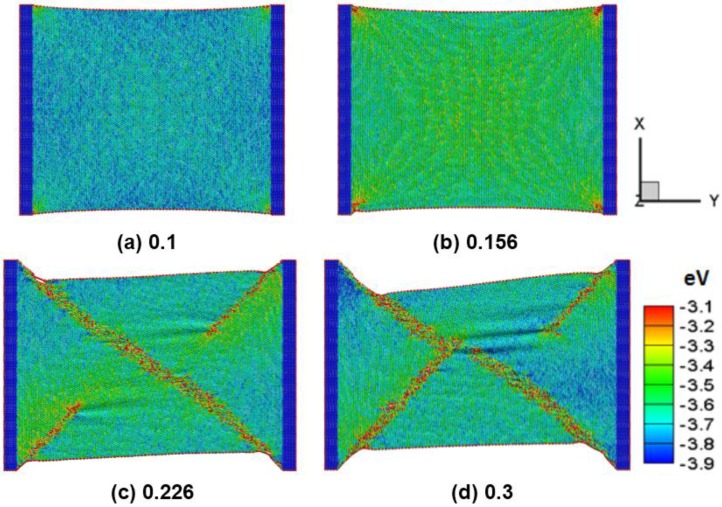
Deformation of an armchair silicene sheet at different tensile strains of (**a**) 0.1; (**b**) 0.156; (**c**) 0.226; and (**d**) 0.3.

**Figure 2:**
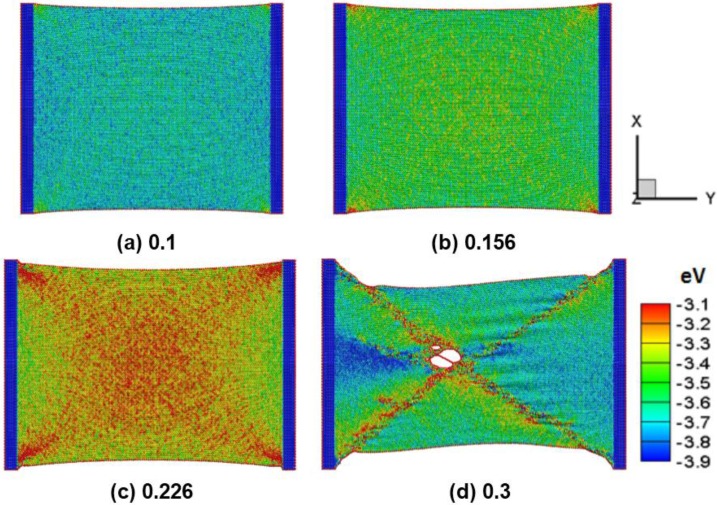
A deformation snapshot of a zigzag silicene sheet at different tensile strains of (**a**) 0.1; (**b**) 0.156; (**c**) 0.226; and (**d**) 0.3.

Figure 1a–d shows a snapshot of an armchair silicene sheet at 300 K under different tensile strains of 0.1, 0.156, 0.226, and 0.3, respectively. Figure 1a shows the armchair silicene sheet exhibiting the stable elastic behavior and the color representing the atomic potential energy variation. The atomistic waves occur, but no warp or wrinkle appears on the tensile silicene surface. By increasing the tensile strain, the armchair silicene has an initial shear deformation around the edge of the silicene sheet under a strain of about 0.156, as depicted in Figure 2b. As the strain increases to 0.226, the high energy band, such as the shear band, occurs in the sheet in the diagonal direction of the nanosheet, as illustrated in Figure 2c. The shear band is about 2–3 nm in width. The local wrinkles are induced by high potential covalently bonded to the interfacial plane. The edge shrinking deformation occurs around the slip band-fixed layer junctions. Stretching the silicene sheet yields wrinklons, which assembles to mimic the complete hierarchy phenomena. A wrinklon occurs and responds to the local transition area, which is needed to merge two wrinkles of wavelength into a larger one. Similar behavior has been discussed with regard to the wrinklons of graphene sheets [18,21]. The fracture gradually becomes large as the strain increases continuously until a strain of 0.3, as shown in Figure 1d. The failure mechanism of silicene sheets under uniaxial tension is attributed to elastic instability, unlike graphene [22]. A crack defect occurs at the high strain edge of the shear band. The sp^3^ bonds of silicon atoms exert a strengthening influence on the wrinkles and stress waves. The wrinklons and waves are observed at the shear band cross center zone of the silicene sheet.

Figure 2a–d shows a snapshot of a zigzag silicene sheet at 300 K under different tensile strains of 0.1, 0.156, 0.226, and 0.3, respectively. Figure 2a shows the zigzag silicene sheets exhibiting the stable symmetric elastic behavior and the ripple of atomic internal energy variation. Figure 2b,c shows the ripples and stress waves being enhanced by the increasing tensile strain, and the silicene has a high strength to bond elongation in the zigzag direction. When the subject strain of 0.3 is enough for large stretching deformation, as shown in Figure 2d, the waviness and shear band distortions occur around the center of the sheet. The complicated crack defects enhance the chain—similar to the fracture of silicon bond nanostructures. The bond breaking and fragmentation of the silicene sheet yield the tensile failure. This tensile failure process was in agreement with a previous study using density functional theory (DFT) calculations [23]. Wu et al. [23] studied dissociative adsorption of a H_2_ molecule on silicene with different tensile strains via DFT calculations. They found that the biaxial strain reached the critical value of about 12%, above which the structure of silicene after hydrogenation would be destroyed [23]. By comparing the different critical strains between DFT and this present result, similar results suggest both structures of silicene would be destroyed. This relative discrepancy is a result of the scale and deformation differences between DFT and MD.

**Figure 3:**
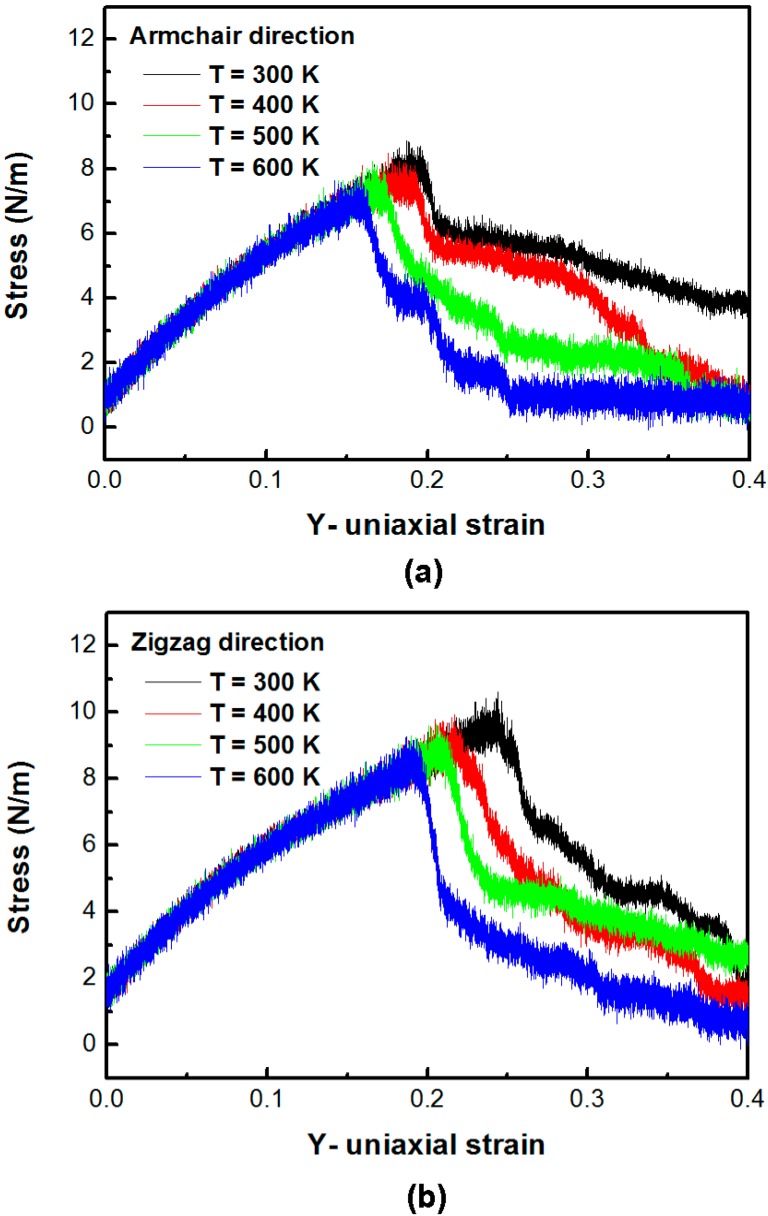
Stress–strain plot of the silicene sheet under uniaxial in (**a**) the armchair direction and (**b**) the zigzag direction at different temperatures.

Figure 3a,b shows the stress–strain plot of the silicene sheet under uniaxial tension in the armchair direction and the zigzag direction at different temperatures, respectively. The average in-plane stresses of the silicene sheet in the armchair direction at temperatures 300, 400, 500, and 600 K is 8.85, 8.50, 8.26, and 7.79 N/m, respectively. The corresponding strains at the maximum stress at temperatures 300, 400, 500, and 600 K are 0.187, 0.176, 0.166, and 0.159, respectively. The average in-plane stresses of the silicene sheet in the zigzag direction at temperatures 300, 400, 500, and 600 K are 10.62, 9.92, 9.64, and 9.27 N/m, respectively. The corresponding strains at the maximum stress at temperatures 300, 400, 500, and 600 K are 0.244, 0.217, 0.205, and 0.187, respectively. The maximum in-plane stress and the corresponding critical strain of the armchair and the zigzag silicene sheets at 300 K are 8.85 and 10.62 N/m, and 0.187 and 0.244, respectively. The calculated effective modulus *E* of the silicene sheet in the armchair and the zigzag directions are 42.21–44.02 and 40.26–41.26 N/m, respectively. The improved mechanical properties can be calculated in a silicene sheet yielded in the zigzag direction compared with the tensile loading in the armchair direction. This is probably due to the chiral effect. This observation is similar to the results of graphene obtained by previous researchers [24,25].

**Figure 4:**
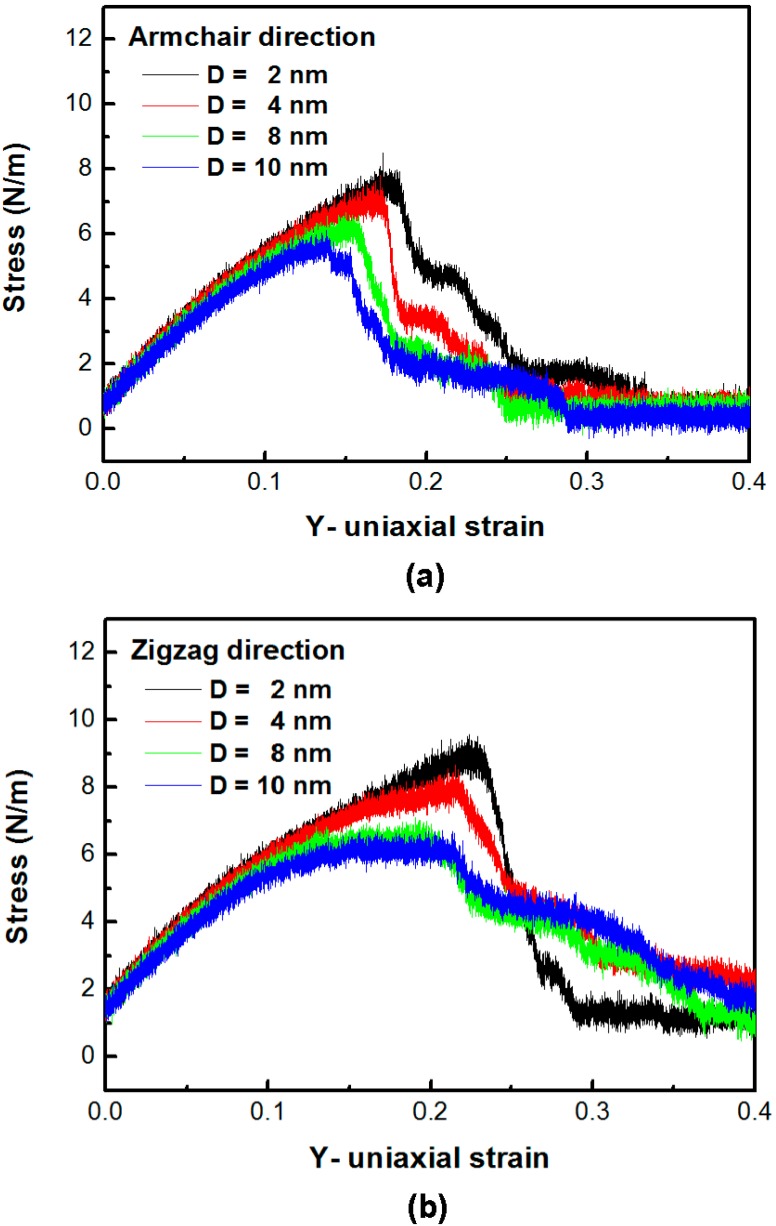
The stress–strain curves in (**a**) the armchair and (**b**) the zigzag silicene under uniaxial tensile strain. The tensile velocity is 10 m/s at 300 K.

Figure 4a,b depicts the in-plane stress–strain curves of the silicene in the armchair and the zigzag directions with different circular hole sizes subject to tension at 300 K, respectively. When the hole size of the armchair and the zigzag silicene increases, the peak stress and the corresponding critical strain decreases. Additionally, the strength of the silicene sheet decreases as the crack hole increases.

**Figure 5:**
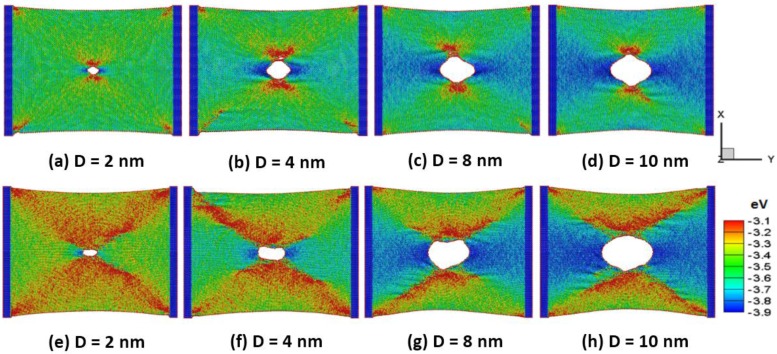
Snapshots of (**a**–**d**) the armchair and (**e**–**h**) the zigzag silicene sheets at uniaxial peak tensile loading at a temperature of 300 K.

Figure 5a,b shows the snapshots of (a–d) the armchair and (e–h) the zigzag silicene sheets with different circular holes of 2, 4, 8 and 10 nm in diameter (D) at a temperature of 300 K at the peak stress, respectively. The hole of the silicene in the armchair direction results in lower waviness and distortions than that of the silicene sheet in the zigzag direction. The dynamic stress waves interact with the hole and the corner of the sheet. The strain induced stress-wave propagation plays an important role in the silicene surface. The higher potential energy zone occurs around the lateral side of the hole under the tensile strain. This is due to the lateral shrinkage of the sheet. Zhao et al. [26] found out the silicene structure failed due to the instability of the low bulked structure based on density functional theory calculations.

**Figure 6:**
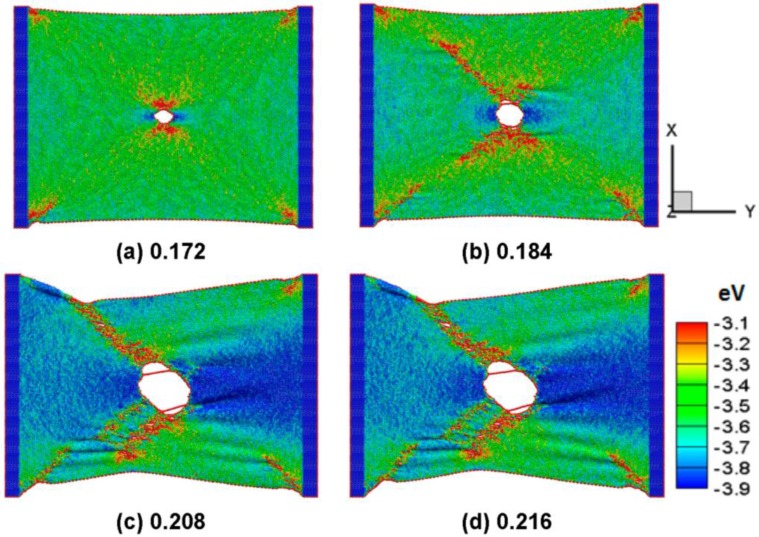
The hole defect diameter is 2.0 nm in the armchair silicene. The colors show the potential energy distribution. The tensile test shows at the strain of (**a**) 0.172; (**b**) 0.184; (**c**) 0.208; and (**d**) 0.216 under a tensile velocity of 10 m/s.

Figure 6a–d shows the snapshots of the silicene nanoribbons in the armchair direction with a circular hole of 2 nm in diameter at a temperature of 300 K at different strains of 0.172, 0.184, 0.208 and 0.216, respectively. The corresponding strains at the maximum stress of pure and defective silicene nanoribbons in the armchair direction at a temperature of 300 K are 0.187 and 0.173, respectively. In Figure 6a, it can be seen that the hole crack is not easily to propagate completely before the critical strain of 0.172. The crack moves quickly after the critical strain, which is accompanied by a large amount of local plastic deformation along the diagonal shear band. The dissipation potential energy decreases with increasing strain due to the warping and ripples inducing the bond elongation at the shear band to release the concentration stress around the hole crack. The stress concentration takes place at the reentrant corner of the silicene sheet. Local stress and potential energy around the hole cavity decrease at larger strains, and propagate and expand along the shear zone, as shown in Figure 6b. Figure 6c,d shows that the failure and the wave shock of the interactions among the nanosheet atoms increased at a higher fracture process.

**Figure 7:**
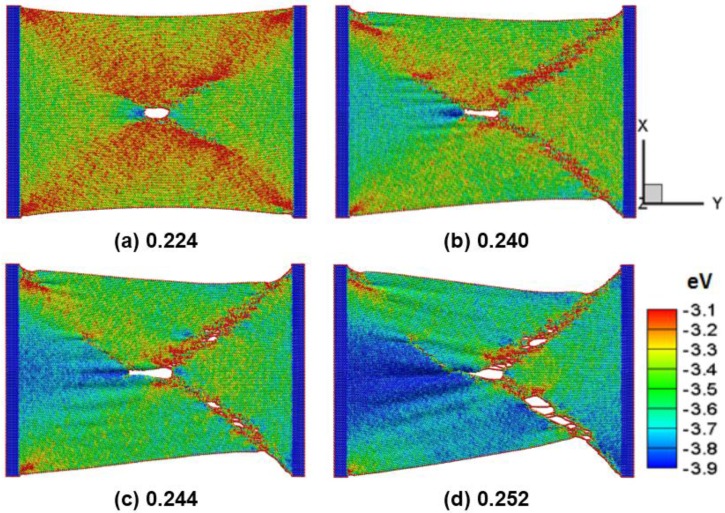
The defect radius is 1.0 nm in zigzag silicene. The colors show the potential energy distribution. The tensile test shows strains of (**a**) 0.224, (**b**) 0.24, (**c**) 0.244, and (**d**) 0.252 in the velocity of 10 m/s.

Figure 7a–d shows the snap of the silicene nanoribbons in the zigzag direction with a circular hole of 2 nm in diameter at a temperature of 300 K at different strain of 0.224, 0.240, 0.244, and 0.252, respectively. The maximum strain at the peak tensile force for pure and crack silicene sheets is 0.244 and 0.224, respectively. The results show that the observed different circular holes vary between the armchair and the zigzag directions. The crack becomes longer but thinner along the tensile direction. The crack propagation originates from the edges and the rapid failure of the silicene sheet. The phenomenon is similar to the previous study on the failure stress and strain of graphene sheets [27].

**Figure 8:**
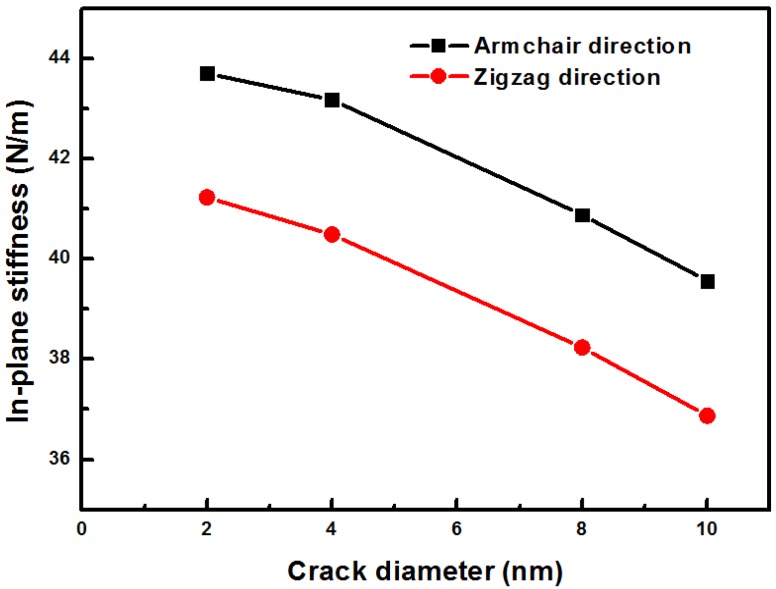
In-plane stiffness using a linear fit of the stress–strain data from the tensile strains of 0.04–0.08.

Figure 8 shows the in-plane stiffness of silicene in the armchair and the zigzag directions. When the hole increases, the stiffness of the sheets decreases. The stiffness of the silicene sheet with a hole in the armchair direction has a high value than that in the zigzag direction. This behavior can be explained by the bond length and the bond angle of the applied tensile strain. When the tension is applied to the silicene in the armchair direction, the bond in the armchair direction stretches parallel to the tension direction. The bond length monotonically increases with increasing strain, but the bond length non-monotonically increases in the zigzag direction stretch. This result is in agreement with density functional theory calculations by Yang et al. [6].

## 4. Conclusions

In summary, MD simulations were performed to examine the mechanical properties of the silicene sheet in the armchair and the zigzag directions at different temperatures subject to tensile loading. The results show that the bond breaking and fragmentation of the silicene sheet yields tensile failure. Improved mechanical properties can be calculated in a silicene sheet yielded in the zigzag direction compared with the tensile loading in the armchair direction. The maximum in-plane stress and the corresponding strain of the silicene sheet decrease as the system temperature increases.

## Figures and Tables

**Table 1:** Comparison of lattice parameters with other models of modified embedded atom method (MEAM) and density functional theory (DFT).

References	Lattice Constant $d_{0}$ (Å)	Bond Length $r_{0}$ (Å)	Bond Angle $\theta_{0}$ (°)	Buckling Height $D_{0}$ (Å)
The present simulation	4.00	2.32	117.98	0.38
MD simulations with MEAM potential [17]	4.05	2.5	-	0.85
MD simulations with Tersoff Potential [18]	3.932	2.313	116.4	0.4444
DFT [19]	3.83	2.25	116.4	0.44
DFT [20]	3.865	2.277	116.134	0.454
